# Central Glossary

**Published:** 2000

Agonist: An agent that mimics the actions or effects of another agent (e.g., a compound that mimics the effects of a *neurotransmitter* and activates that neurotransmitter’s *receptor*).
Allele: Different forms of a *gene*. Different alleles for a gene serve the same function (e.g., code for a *protein* that affects a person’s eye color) but may result in different *phenotypes* (e.g., blue eyes or brown eyes).
Antagonist: An agent that blocks or reverses the effects of another agent (e.g., a compound that blocks the effects of a *neurotransmitter* or inactivates that neurotransmitter’s *receptor*).
Antioxidant: A substance (e.g., glutathion or vitamin E) that inhibits *oxidation*.
Association study: A study that analyzes whether a certain *DNA marker* or *allele* is inherited with a particular disease or disorder (e.g., alcoholism) more frequently than would be expected in the general population; the marker or allele does not necessarily play a role in the development of the disease, however, but may just be located adjacent to a gene that contributes to the disease.
Candidate gene: A *gene* that has been implicated in causing or contributing to the development of a particular disease.
Cardiomyopathy: Any disease that affects the structure and function of the heart.
Catecholamine: One of a group of physiologically active substances with various roles in the functioning of the nervous system; also helps regulate heart functioning.
Cerebellum: A brain structure at the base of the brain that is involved in the control of muscle tone, balance, and sensorimotor coordination.
Cerebral cortex: The intricately folded outer layer of the brain, composed of nerve-cell bodies (gray matter); contains areas for processing sensory information and for controlling motor functions, speech, higher cognitive functions, emotions, behavior, and memory.
Chromosomes: Threadlike molecules in the cell that consist of *DNA* and *protein* and contain most of the cell’s *genes*. Humans have 46 chromosomes arranged in 23 pairs.
Collagen: The major *protein* component of fibrous connective tissue (e.g., tendons and ligaments); also found in scar tissue.
Congenic strain: A strain of animals, such as mice, in which a *DNA* segment from one strain has been transferred (or introgressed) into the *genome* of a host animal (typically an inbred animal). In a set of congenic strains, the animals are identical with respect to the vast majority of their genetic material and differ only in the introgressed DNA segment.
Cytokine: A molecule that regulates cellular interactions and cellular functions. Cytokines are produced and secreted by a variety of cells, including immune cells.
Cytosol: The portion of the cell that contains soluble materials.
Cytoskeleton: Fiber-like structures within a cell that help give the cell its shape and stability.
Dendrite: A type of thin, branched nerve cell fiber that extends from the *neuron’s* body to receive information from other neurons.
Diastole: The time period between two contractions of the heart during which blood enters the relaxed heart chambers from the lungs and from the systemic circulation.
DNA: The abbreviation for deoxyribonucleic acid, the molecule that encodes the genetic information in all organisms except some viruses. DNA molecules usually consist of two strings of nucleotides. DNA is a component of *chromosomes*.
DNA marker: A *DNA* region with a known sequence.
Endogenous opioids: A group of brain chemicals that bind to opiate *receptors* in the brain, resulting in such effects as euphoria and pain relief; also contribute to alcohol *reinforcement*.
Endophenotype: A *phenotype* that is not immediately visible but may contribute to the susceptibility to develop a particular behavior or syndrome.
Endothelium: A layer of cells lining the inner wall of a blood vessel.
Endotoxin: A molecule in the cell wall of many bacteria, including many bacteria in the intestine. Endotoxins are released and may enter the bloodstream when the bacteria die; they can cause fever, chills, shock, and other symptoms of infection.
Epithelium: The tissue layer lining the internal and external organs of the body as well as the blood vessels, body cavities, and glands.
Excitation-contraction (E-C) coupling: The process through which the electrical excitation of a muscle cell membrane that occurs in response to a nerve signal leads to the contraction of the muscle cell.
Extinction: The “un-learning” of a previously learned behavior (e.g., pressing a lever in order to receive alcohol); can be achieved by withholding the reward (i.e., alcohol) that was associated with the learned behavior.
Fatty acids: Molecules that constitute components of fat molecules (i.e., lipids)
Fibrosis: Formation of scar tissue.
Forward genetics: Strategy in genetic research by which investigators first introduce mutations into the *DNA* and then assess the result of those mutations on the function of a cell or an organism (e.g., whether the cell or organism loses certain functions or characteristics).
Free iron: Iron molecules with a low molecular weight that can form bonds with other molecules (e.g., *proteins*).
Gene: A string of *nucleotides* that directs the synthesis of a *protein*.
Gene expression: The process of converting the genetic information encoded in the *DNA* into the final *gene* product (i.e., a *protein*).
Geneticmarker: A segment of *DNA* with an identifiable physical location on a *chromosome* whose inheritance can be followed.
Genome: The sum of all *genes* in an individual organism.
Genotype: The genetic makeup of an individual organism.
Germ cell: A cell responsible for reproduction (i.e., the sperm cells in males and the eggs in females) or its precursors. Germ cells contain only half the number of *chromosomes* as *somatic cells*.
Germ-line transformation: A technique for introducing genetic changes into the *germ cells* (i.e., eggs or sperm cells) of an organism so that these changes are passed on to the offspring.
Hepatitis: Inflammation of the liver.
Hepatocyte: The principal cell type found in the liver; its many functions include bile production, *protein* synthesis, detoxification, and nutrient storage.
Hippocampus: A curved ridge found within the cerebral hemisphere that functions in consolidating new memories.
Inbred strain: A virtually genetically identical group of organisms derived by inbreeding among a limited number of ancestors. An inbred strain of mice is like a population of identical twins.
In vitro: Latin for “in glass,” as in a test tube. An in vitro test is done in the laboratory using preparations of isolated cells, tissues, or organs.
Knockout/knock-in mice: Mice in which a *gene* has been deleted (knockedout) or *mutated* (knocked-in) in both the *somatic* and the *germ cells* so that the animals produce no functional gene product.
Kupffer cell: A *phagocytic cell* in the liver that removes bacteria and other foreign organic substances from the blood by ingesting them.
Linkage study: A study that looks for *genes* or *alleles* that are necessary or sufficient for the development of a particular disorder.
Locus, loci: A specific location(s) on a *chromosome*.
Microsome: Small vesicles derived from one of the cell’s *organelles* (i.e., the endoplasmatic reticulum) when cells are forcibly broken up.
Mitochondria: *Organelles* that generate the energy (e.g., adenosine triphosphate [ATP]) required for many cell functions.
Mitogen: A molecule that stimulates cell division.
Mutagen: Any substance that can induce a *mutation*.
Mutation: A change, deletion, or rearrangement in the *DNA* sequence that may lead to the synthesis of an altered *protein* or to a totally inactive *gene* incapable of producing a protein.
Myocardium: A thick layer of uniquely constructed and arranged muscle cells that forms the bulk of the heart wall.
Myocyte: A muscle cell.
Necrosis: Death of one or more cells or of portions of tissue resulting from adverse conditions or changes in the cell’s environment.
Neuron: A nerve cell.
Neurotransmitter: A chemical messenger released by an excited or stimulated *neuron*. After release, neurotransmitters travel across a *synapse* and bind to docking molecules (i.e., *receptors*) on an adjacent neuron or muscle cell, usually triggering a series of chemical and electrical changes in the second cell.
Neuroadaptive changes: Changes in nerve cell *gene expression* and in the connections among nerve cells that occur in response to environmental changes (e.g., chronic exposure to alcohol or other drugs).
Neuropeptide: A small molecule that can regulate nerve cell function and is made up of amino acids.
Neurotransmitter: A chemical messenger released by a nerve cell that transmits a nerve signal from that cell to a neighboring nerve cell.
Organelle: A functional component of a cell (e.g., a *mitochondrion* or the endoplasmic reticulum ); each organelle has its own membrane and specialized function.
Oxidation: A chemical reaction that removes a hydrogen atom from a substance or adds oxygen to it (or both).
Oxidative stress: An imbalance between oxidants and *antioxidants*, leading to excessive *oxidation* and cell damage.
Oxygen radical: Highly reactive, oxygen-containing molecules that cannot exist in a free state for a prolonged period; also called reactive oxygen species.
Paradigm: A specific experimental design or approach.
Pariet al cortex: A subdivision of the *cerebral cortex* involved in controlling higher cognitive functioning and the integration of sensory information.
Perfused organ: An isolated organ that is kept functional by passing a fluid (i.e., medium) through it.
Phagocytic cell: Any type of cell that eliminates foreign substances, microorganisms, or damaged cells by ingesting them.
Phenotype: The observable properties, traits, or physical appearance of an organism resulting from the interaction of the *genotype* with environmental factors.
Polymorphic marker: A *DNA marker* that exists in different forms (i.e., *alleles*) within a test population.
Polymorphism: Occurrence of two or more *alleles* at a high frequency in the population
Primary cell culture: *In vitr*o cultivation of cells that have been newly isolated from an organism.
Promoters: Stretches of *DNA* associated with a specific gene that guide the expression of the gene to specific areas in the brain and “turn on” the expression of the gene.
Prostaglandins: A group of *hormone-like*, unsaturated *fatty acids* that act in exceedingly low concentrations on a variety of organs, regulating, for example, heart function, smooth muscle tone, and nervous system function.
Protein: The product of the genetic information encoded in a *gene*. Proteins are made up of amino acids whose order is dictated by the gene’s nucleotide sequence.
Purkinje cells: Specialized *neurons* in the *cerebellum* that send signals from the cerebellum to other neurons after the cerebellar cortex has processed sensory and motor information from the rest of the nervous system.
Quantitative trait: A trait, or characteristic, that is determined by more than one *gene* and which exists in many different degrees (i.e., is distributed continuously) within a population. Body height is an example of a quantitative trait.
Receptor: A protein that serves as a “docking molecule” for signaling molecules, such as *neurotransmitters* and hormones, and which mediates the actions of those signaling molecules.
Recombinant inbred (RI) strain: An animal strain generated by mating two *inbred strains* and then inbreeding the F_2_ (“grand-child”) generation; in an RI strain, the genetic material from the original inbred strains has been recombined as a result of the *DNA* rearrangement that occurs during the specialized cell division (i.e., meiosis) that results in the production of egg and sperm cells.
Redox state: The ratio of oxidized to reduced reactants in a cell.
Reinforcement: A process in which a response or behavior (e.g., alcohol consumption) is strengthened by the anticipation of a reward (e.g., a feeling of euphoria).
Reverse genetics: Strategy in genetic research by which investigators begin by selecting a specific *gene* and then try to generate a mutant organism that lacks the function of that gene.
Somatic cells: All cells of an organism other than the *germ cells*.
Stellate cell: A star-shaped, droplet-containing cell that serves as the primary storage site for vitamin A compounds and fat molecules; plays a key role in the development of *fibrosis* after being activated.
Synapse: A microscopic gap separating adjacent *neurons* or neurons and *myocytes*.
Thymus: A lymphoid organ near the base of the neck in which *T-lymphocytes* mature.
T-lymphocyte: A type of immune cell that originates in the bone marrow and matures in the *thymus* and which plays a central role in regulating the immune response (e.g., by secreting important *cytokines*).
Transcription factor: A *protein* that binds to the *DNA*, regulating the conversion of genetic information into proteins.
Transformed cell line: A cell line, frequently derived from a tumor and grown *in vitro*, that no longer responds to normal growth control mechanisms and can divide indefinitely.
Transposable element: A small piece of *DNA* that can change its position in an organism’s genetic material; if such a DNA piece by chance integrates into a *gene*, that gene (and the *protein* it encodes) may be altered or even inactivated.
Transposon tagging: The process by which the known *DNA* sequence of a transposable element (i.e., P element) is used as a tag to isolate the genomic DNA located adjacent to the site of transposon integration. This procedure allows the cloning of the *gene* whose function is disrupted by the P element.
Tumor necrosis factor alpha (TNF-α): A *cytokine* produced by a type of immune cell (i.e., macrophages) and which, among other functions, has anticancer effects.
Vesicle: A small, bubble-like component of the *cytosol* that serves to store various types of molecules (e.g., *nerotransmitters*).

